# COGNITIVE FUNCTION AND LIFE SATISFACTION IN LATER LIFE: IS EMOTIONAL WELL-BEING A PATHWAY?

**DOI:** 10.1093/geroni/igad104.2621

**Published:** 2023-12-21

**Authors:** Brittany McFeeley, Changmin Peng, Jeffrey Burr

**Affiliations:** University of Massachusetts Boston, Boston, Massachusetts, United States; University of Massachusetts, Boston, Boston, Massachusetts, United States; University of Massachusetts Boston, Boston, Massachusetts, United States

## Abstract

Cognitive impairment and dementia represent major public health concerns globally. Life satisfaction measures capture an important element of subjective well-being in later life. This study examines the association between cognitive function and life satisfaction along with the mediating effects of loneliness and depression among older adults. While the study of life satisfaction and cognitive function have received considerable attention among gerontologists, we know relatively little about whether emotional well-being represents a pathway between cognitive function and life satisfaction. To address our research questions, we employed nationally representative data from the 2018 Health and Retirement Study, including information from the Leave Behind Questionnaire, focusing on community dwelling respondents aged 65 and older (N=3,173). Cognitive function is measured with the Telephone Interview for Cognition Status instrument and life satisfaction is based on the Diener Life Satisfaction Scale. Depression is measured by the number of depressive symptoms (CESD-8) and loneliness is based on the UCLA Loneliness Scale. Results from our linear regression models show that cognitive function is positively associated with life satisfaction (B=0.012, p=.028), while depressive symptoms (B=-0.261, p<.001) and loneliness are negatively associated with life satisfaction (B=-1.290, p<.001). Each measure of emotional well-being mediates the cognitive function-life satisfaction relationship, supporting our hypothesis. Future research based on longitudinal designs should evaluate whether other mediators help explain this relationship, including the structural and functional domains of social networks. Social network characteristics may combine with emotional well-being to provide an improved understanding of cognitive function and subject well-being.

